# Efficacy and safety of tislelizumab plus lenvatinib as first-line treatment in patients with unresectable hepatocellular carcinoma: a multicenter, single-arm, phase 2 trial

**DOI:** 10.1186/s12916-024-03356-5

**Published:** 2024-04-23

**Authors:** Li Xu, Jinzhang Chen, Chang Liu, Xiaoling Song, Yanqiao Zhang, Haitao Zhao, Sheng Yan, Weidong Jia, Zheng Wu, Yabing Guo, Jiayin Yang, Wei Gong, Yue Ma, Xiaobo Yang, Zhenzhen Gao, Nu Zhang, Xin Zheng, Mengyu Li, Dan Su, Minshan Chen

**Affiliations:** 1 Department of Hepatobiliary Oncology, Sun Yat-Sen University Cancer Center, State Key Laboratory of Oncology in South China, Collaborative Innovation Center for Cancer Medicine, 651 Dongfeng Road East, Guangzhou, 510060 China; 2 Department of Infectious Diseases and Hepatology, State Key Laboratory of Organ Failure Research, Guangdong Provincial Key Laboratory of Viral Hepatitis Research, Nanfang Hospital, Southern Medical University, Guangzhou, China; 3 Division of Liver, Department of General Surgery, West China Hospital, Sichuan University, Chengdu, China; 4 Department of Minimal Invasive Surgery, Shangjin Nanfu Hospital, Chengdu, China; 5 Department of General Surgery and Laboratory of General Surgery, Xinhua Hospital, Shanghai Jiao Tong University School of Medicine, Shanghai Key Laboratory of Biliary Tract Disease Research, Shanghai, China; 6 Department of Gastrointestinal Medical Oncology, Harbin Medical University Cancer Hospital, Harbin, China; 7 Department of Liver Surgery, Peking Union Medical College Hospital, Beijing, China; 8 Department of Hepatobiliary and Pancreatic Surgery, Second Affiliated Hospital, Zhejiang University School of Medicine, Hangzhou, China; 9 Surgery Department, Anhui Provincial Hospital, Hefei, China; 10 Department of Hepatobiliary Surgery, The First Affiliated Hospital of Xi’an Jiaotong University, Xi’an, China; 11 Department of General Surgery, Liver Transplant Center, Transplant Center, West China Hospital, Sichuan University, Chengdu, China; 12 Medical Affairs, BeiGene (Beijing) Co., Ltd., Beijing, China; 13 Global Statistics and Data Science, BeiGene (Shanghai) Co., Ltd., Shanghai, China

**Keywords:** Immunotherapy, Anti-PD1 antibody, Combination therapy, Multikinase inhibitor, Hepatocellular carcinoma, Clinical trial, Tislelizumab, Lenvatinib

## Abstract

**Background:**

Lenvatinib is widely used in treatment of unresectable hepatocellular carcinoma (uHCC), but the benefit of its combination with immunotherapy needs to be verified. This study evaluated the efficacy and safety of tislelizumab plus lenvatinib in systemic treatment-naïve patients with uHCC.

**Methods:**

In this multicenter, single-arm, phase 2 study, systemic treatment-naïve patients with uHCC received tislelizumab 200 mg every three weeks plus lenvatinib (bodyweight ≥ 60 kg: 12 mg; < 60 kg: 8 mg; once daily). Dose-limiting toxicities (DLTs) were evaluated in safety run-in phase to determine whether to enter the expansion phase. The primary endpoint was objective response rate (ORR) assessed by independent review committee (IRC) per Response Evaluation Criteria in Solid Tumors, version 1.1 (RECIST v1.1). Based on Simon’s two-stage design, > 6 responders were needed in stage 1 (*n* = 30) to continue the study, and ≥ 18 responders were needed by the end of stage 2 (*n* = 60) to demonstrate statistical superiority to a historical control of lenvatinib monotherapy.

**Results:**

Sixty-four patients were enrolled. No DLTs were reported. The study achieved statistical superiority (*p* = 0.0003) with 23 responders assessed by IRC per RECIST v1.1 in the first 60 patients of the efficacy evaluable analysis set (*n* = 62). After a median follow-up of 15.7 months, confirmed ORR and disease control rate were 38.7% (24/62, 95% confidence interval [CI], 26.6–51.9) and 90.3% (56/62, 95% CI, 80.1–96.4), respectively. Median progression-free survival was 8.2 months (95% CI, 6.8–not evaluable). Overall survival rate at 12 months was 88.6% (95% CI, 77.7–94.4). Grade ≥ 3 treatment-related adverse events occurred in 18 (28.1%) patients.

**Conclusions:**

Tislelizumab plus lenvatinib demonstrated promising antitumor activity with favourable tolerability as first-line therapy for patients with uHCC.

**Trial registration:**

ClinicalTrials.gov (NCT 04401800).

**Supplementary Information:**

The online version contains supplementary material available at 10.1186/s12916-024-03356-5.

## Background

Hepatocellular carcinoma (HCC) is the sixth most common cancer and the third leading cause of cancer-related deaths worldwide [1]. The majority of HCC (approximately 72%) are diagnosed in Asia, with hepatitis B virus (HBV) infection being the most common etiology of HCC [2, 3]. Despite advancements in early detection, most patients with HCC still present with advanced disease, which limits the opportunity for radical treatment.

Globally, tyrosine kinase inhibitors (TKIs) sorafenib and lenvatinib are recommended as first-line treatments for unresectable HCC (uHCC) [4, 5]. However, the clinical benefits with TKIs were limited due to the unsatisfying objective response rates (ORRs) (for instance, 2% of sorafenib in SHARP study, 18.8% of lenvatinib in REFLECT study) [4, 5]. More therapeutic options are needed to expand the patient population that could benefit from TKIs. In recent years, immuno-oncology therapies, such as immune-checkpoint inhibitors (ICIs), have reshaped the treatment landscape for advanced HCC. Combining an ICI with a TKI is a promising combination strategy as TKIs may have effects on the vascular endothelial growth factor receptor (VEGFR) and other kinases that may modulate the activity of ICIs [6–8]. The global LEAP-002 study investigated the efficacy and safety of lenvatinib plus pembrolizumab as a first-line treatment in uHCC patients [9]. Though it failed to achieve its dual primary endpoints of overall survival (OS) and progression-free survival (PFS) in the global intent-to-treatment population, recent subgroup analysis revealed encouraging median OS (26.3 vs 22.4 months; hazard ratio [HR] 0.727; 95% confidence interval [CI], 0.552–0.958) and PFS (8.3 vs 6.5 months; HR 0.710; 95% CI, 0.556–0.907) benefit trend compared with lenvatinib alone in Asian population [10], suggesting potential benefits of this combination strategy in Asian patients with a high incidence of HBV-related etiology. Furthermore, a retrospective real-world study in China reported that lenvatinib plus programmed death-1 (PD-1) inhibitors treatment showed considerable overall survival of 17.8 months in uHCC patients not restricted to first-line therapy [11]. Taken together, these findings suggest that lenvatinib plus a PD-1 inhibitor is a promising treatment strategy for uHCC patients in China. However, there is no published study prospectively exploring the efficacy and safety of tislelizumab in combination with lenvatinib in the first-line setting.

Tislelizumab is a monoclonal antibody with high affinity and binding specificity for PD-1, which was designed to minimize binding to Fcγ receptors on macrophages to limit antibody-dependent cellular phagocytosis, a potential mechanism contributing to anti-PD-1 therapy resistance [12]. In a global randomized phase 3 study (RATIONALE-301), single-agent tislelizumab demonstrated clinically meaningful OS benefit that was non-inferior to sorafenib (median OS, 15.9 vs 14.1 months; HR 0.85; [95.003% CI, 0.71 to 1.02]) with a favourable safety profile as a first-line treatment option for patients with uHCC [13]. We performed a phase 2 study (BGB-A317-211) to explore the efficacy and safety of tislelizumab in combination with lenvatinib as first-line treatment in Chinese patients with unresectable locally advanced or metastatic HCC.

## Methods

### Study design and participants

BGB-A317-211 was a prospective, multicenter, open-label, single arm, phase 2 study evaluating tislelizumab plus lenvatinib as first-line treatment in patients with uHCC, conducted in 9 sites across China. This study consisted of a safety run-in phase and an expansion phase (Additional file 1: Fig. S1). Study subjects received intravenous tislelizumab 200 mg on day 1 for a 21-day treatment cycle, in combination with lenvatinib 12 mg (body weight ≥ 60 kg) or 8 mg (body weight < 60 kg) orally taken once daily. The treatment dosage was chosen referring to the phase 1b study KEYNOTE-524, which showed manageable safety profile of anti-PD-1 antibody (pembrolizumab) plus lenvatinib with the combination dose following prescription instructions of each drug [14]. During the study design, tislelizumab had been approved in China at a dose of 200 mg intravenously every 3 weeks, however the safety profile of its combination with lenvatinib in HCC had not been evaluated before, therefore, a safety run-in phase (Additional file 2: Supplemental methods) was designed to further ensure patients’ tolerance of the present combination.

Treatment was continued until immune confirmed disease progression (iCPD) assessed by immune Response Evaluation Criteria in Solid Tumors (iRECIST), development of unacceptable toxicity, death, withdrawal of consent, or completion of 12 months treatment. Patients who completed 12 months of treatment without iCPD and were deemed to still benefit from the study treatment based on investigator assessment were allowed to continue receiving tislelizumab.

Eligible patients were aged 18–70 years with histologically or cytologically confirmed unresectable locally advanced or metastatic HCC who had no prior systemic therapy. Key inclusion criteria included a Barcelona Clinic Liver Cancer (BCLC) Stage C or B disease, Child–Pugh A classification for liver function, at least one measurable lesion as defined by Response Evaluation Criteria in Solid Tumors version 1.1 (RECIST v1.1), Eastern Cooperative Oncology Group (ECOG) performance status score of ≤ 1, no tumor thrombus involving the main trunk of the portal vein or inferior vena cava, and a life expectancy of ≥ 3 months. Patients were excluded if they had any known brain or leptomeningeal metastases. Detailed inclusion and exclusion criteria are available at ClinicalTrials.gov (NCT 04401800).

The study was approved by the institutional review board or ethics committee for all participating centers and was conducted in accordance with the principles of the Declaration of Helsinki. Written informed consent was obtained from all participants.

### Outcomes and Clinical Assessments

Tolerability and safety of the combination were initially assessed by evaluating dose-limiting toxicities (DLTs) based on hematology and non-hematology toxicities in safety run-in phase (Additional file 2: Supplemental methods). The primary endpoint was the confirmed ORR by RECIST v1.1 per independent review committee (IRC). ORR was defined as the proportion of patients with complete response (CR) or partial response (PR) as their best overall response. Secondary endpoints included safety and tolerability, IRC-assessed ORR per mRECIST and iRECIST, IRC-assessed duration of response (DoR; time between first CR or PR and disease progression [PD] or death), disease control rate (DCR; proportion of patients with a best overall response of CR, PR or stable disease [SD]), and PFS (time from first dose of study medication to PD or death) per RECIST v1.1, mRECIST and iRECIST. Investigator-assessed ORR, DoR, DCR, and PFS per RECIST v1.1, mRECIST and iRECIST were also secondary endpoints. OS was an exploratory endpoint, which was defined as the time from first dose of study medication to death.

Tumor radiographic assessments were performed at baseline, every 6 weeks in the first year of treatment, and every 9 weeks thereafter. Adverse events (AEs) were classified based on Medical Dictionary for Regulatory Activities (MedDRA) Version 25.0 and graded according to National Cancer Institute Common Terminology Criteria for Adverse Events (NCI-CTCAE) version 5.0.

### Statistical analyses

An overall sample size of 60 patients was planned to test the statistical hypothesis for this study. Based on Simon’s two-stage design, the study had about 95% power to detect a statistically significant difference of ORR (assessed based on RECIST v1.1 by IRC) in tislelizumab plus lenvatinib (expected to be 40%, referring to the ORR of 36% for lenvatinib plus pembrolizumab in the study KETNOTE-524 [14]), compared with a historical control of 18.8% (referring to the ORR of lenvatinib monotherapy from REFLECT study [5]) with 1-sided alpha as 0.025. Within the first 30 patients (including the 6 patients from the safety run-in phase and 24 patients from the expansion phase) in the efficacy evaluable analysis set (EAS), > 6 responders (CR or PR) were needed in the interim analysis (*n* = 30) for the study to continue. If within the final 60 patients in the EAS, ≥ 18 responders were observed, statistical superiority to a historical control of 18.8% would be claimed under the settings. The sample size was estimated using R 4.1.2 (Additional file 2: Supplemental methods). Considering potential dropouts and actual enrolment conducted simultaneously in multiple sites, the study allowed to include no more than 6 additional patients.

Safety analyses were performed in the safety analysis set (SAS), which included all patients who received at least one dose of tislelizumab or lenvatinib. The EAS included all patients from the SAS who had measurable disease at baseline (per RECIST v1.1) and at least one evaluable post-baseline tumor assessment unless treatment was discontinued for disease progression or death before the first assessment. All efficacy analyses were conducted in the EAS.

Descriptive statistics were used to summarize the data. The continuous and categorical variables were expressed as median (range) and number (percentage), respectively, unless otherwise specified. ORR and DCR were calculated with 95% CIs estimated using the Clopper-Pearson method. Time-to-event variables (DoR, PFS and OS) were estimated using the Kaplan–Meier method, and median values were presented with 95% CIs calculated by the Brookmeyer-Crowley method. PFS and OS rates at 6 months or 12 months were calculated using the Kaplan–Meier method, and their 95% CIs were calculated by the Greenwood formula. All statistical analyses were performed using SAS version 9.4 (SAS Institute, Cary, NC).

## Results

### Patients and treatment

Between September 4, 2020, and January 7, 2022, a total of 64 patients were enrolled (Additional file 1: Fig. S2), and all these patients received study treatment (SAS, including subjects of safety run-in phase, *n* = 6; and expansion phase, *n* = 58). The median age of patients was 52.5 years (range, 28.0–70.0), 82.8% were male. At study entry, 17 (26.6%) patients were BCLC stage B and 47 (73.4%) were stage C. Child–Pugh score of 5 and 6 were noted in 58 (90.6%) and 6 (9.4%) patients, respectively. Thirty-seven (57.8%) patients exhibited extrahepatic spread, and 7 (10.9%) had macrovascular invasion. Hepatitis B served as the most common etiology of HCC (58/64, 90.6%). Twenty-six (40.6%) cases had a baseline alpha-fetoprotein level of ≥ 400 ng/mL. Most of the patients (73.4%) had experienced disease progression of previous locoregional therapy (Table 1).

**Table 1:** Patient demographics and baseline characteristics

Characteristic	All patients (*n* = 64)
Median age, years (range)	52.5 (28.0–70.0)
Sex	
Male	53 (82.8)
Female	11 (17.2)
Race, Asian	64 (100.0)
Region, mainland China	64 (100.0)
BCLC staging at study entry	
B	17 (26.6)
C	47 (73.4)
ECOG performance score	
0	40 (62.5)
1	24 (37.5)
Child–Pugh score	
5	58 (90.6)
6	6 (9.4)
Extrahepatic spread	37 (57.8)
Macrovascular invasion	7 (10.9)
AFP level	
≥ 400 ng/mL	26 (40.6)
< 400 ng/mL	38 (59.4)
HCC etiology, HBV	
Yes	58 (90.6)
No	6 (9.4)
Prior liver local regional therapy	47 (73.4)
Radio frequency ablation	11 (17.2)
Microwave frequency ablation	4 (6.3)
TACE or TE	31 (48.4)
Hepatic artery infusion chemotherapy	14 (21.9)
Other (including surgery)	5 (7.8)

Data are presented as n (%) unless otherwise indicated. *AFP* Alpha-fetoprotein, *BCLC* Barcelona Clinic Liver Cancer, *ECOG* Eastern Cooperative Oncology Group, *HBV* Hepatitis B virus, *HCC* Hepatocellular carcinoma, *TACE* Transarterial chemoembolization, *TE* Transarterial embolism

As of December 16, 2022, the median follow-up duration was 15.7 months (range, 0.9–27.4). One patient was still receiving study treatment (last cycle of treatment). The median treatment duration of tislelizumab was 11.0 months (range, 0.7–11.9). The median treatment duration of lenvatinib was 11.1 months (range, 0.3–12.1). Overall, 63 (98.4%) patients discontinued study treatment. The primary reason for treatment discontinuation was completion of the planned 12-month study treatment (*n* = 33, 51.6%). In this study, 28 patients continued to receive study treatment after progression per RECIST v1.1, with a median post-progression treatment of 1.5 months (range, 0.1–10.6). During the post-progression treatment period, 6 patients had target lesions shrinkage compared with baseline and had no new lesions, and of which 1 underwent subsequent curative surgery. Data on subsequent anticancer medications during survival follow-up are summarized in Additional file 3: Table S1.

### Safety run-in phase

No DLTs were observed in the first 6 patients of the safety run-in phase. Therefore, the study combination was administered as per the planned dosage consistently throughout the entire study with lenvatinib 12 mg (body weight ≥ 60 kg) or 8 mg (body weight < 60 kg) orally taken once daily and intravenous tislelizumab 200 mg on day 1 for a 21-day treatment cycle.

### Efficacy

There were 23 responders per RECIST v1.1 assessed by IRC in the first 60 patients of the EAS, which was more than the preset threshold of 18 responders based on the Simon’s two- stage design, indicating that statistical superiority of the study combination therapy over historical control lenvatinib monotherapy was achieved (*p* = 0.0003).

**Fig. 1:**
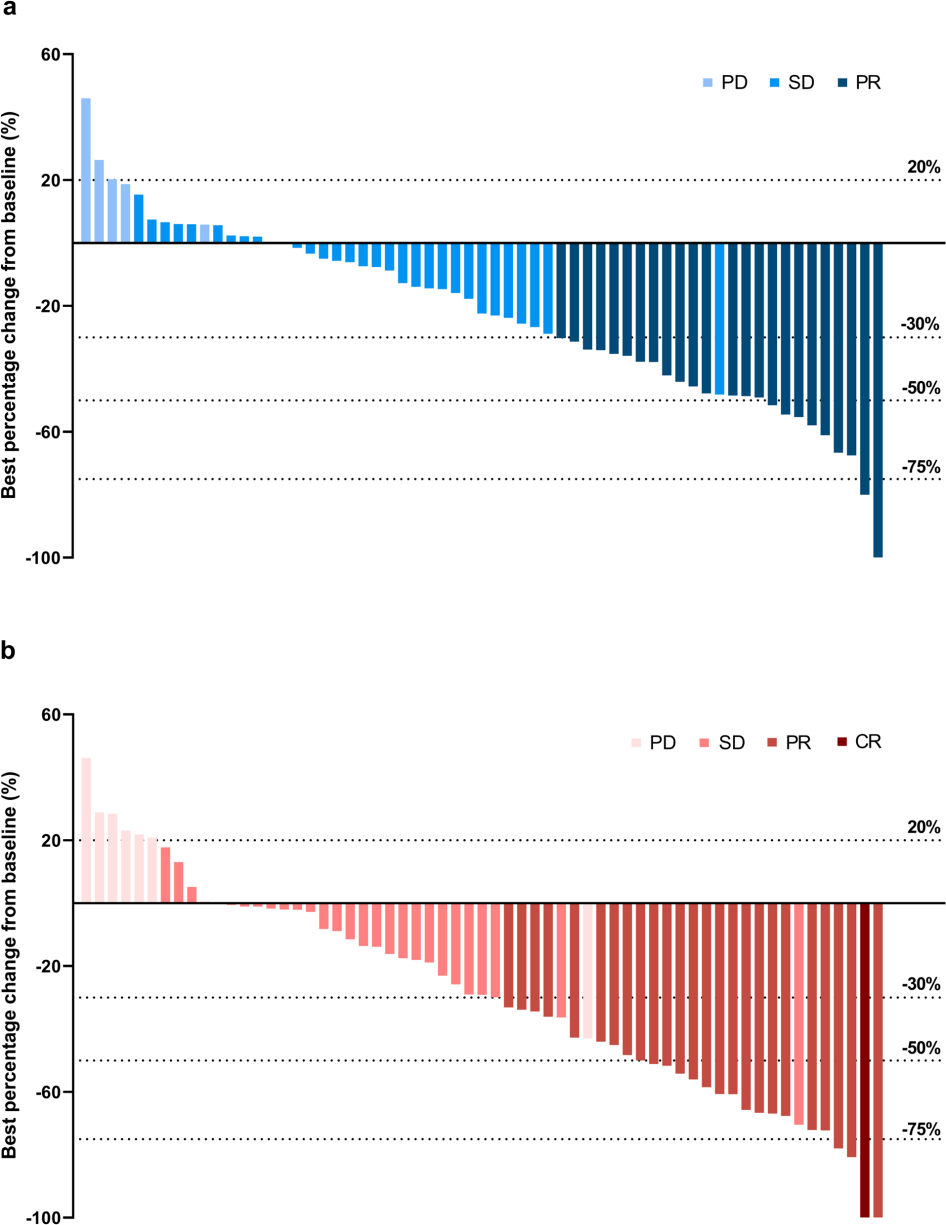
Best percentage change from baseline in sum of diameters of target lesions per RECIST v1.1 by **a** IRC review and **b** Investigator review (*N* = 62). IRC = Independent Review Committee, RECIST = Response Evaluation Criteria in Solid Tumors, PD = progressive disease, SD = stable disease, PR = partial response, CR = complete response

**Fig. 2:**
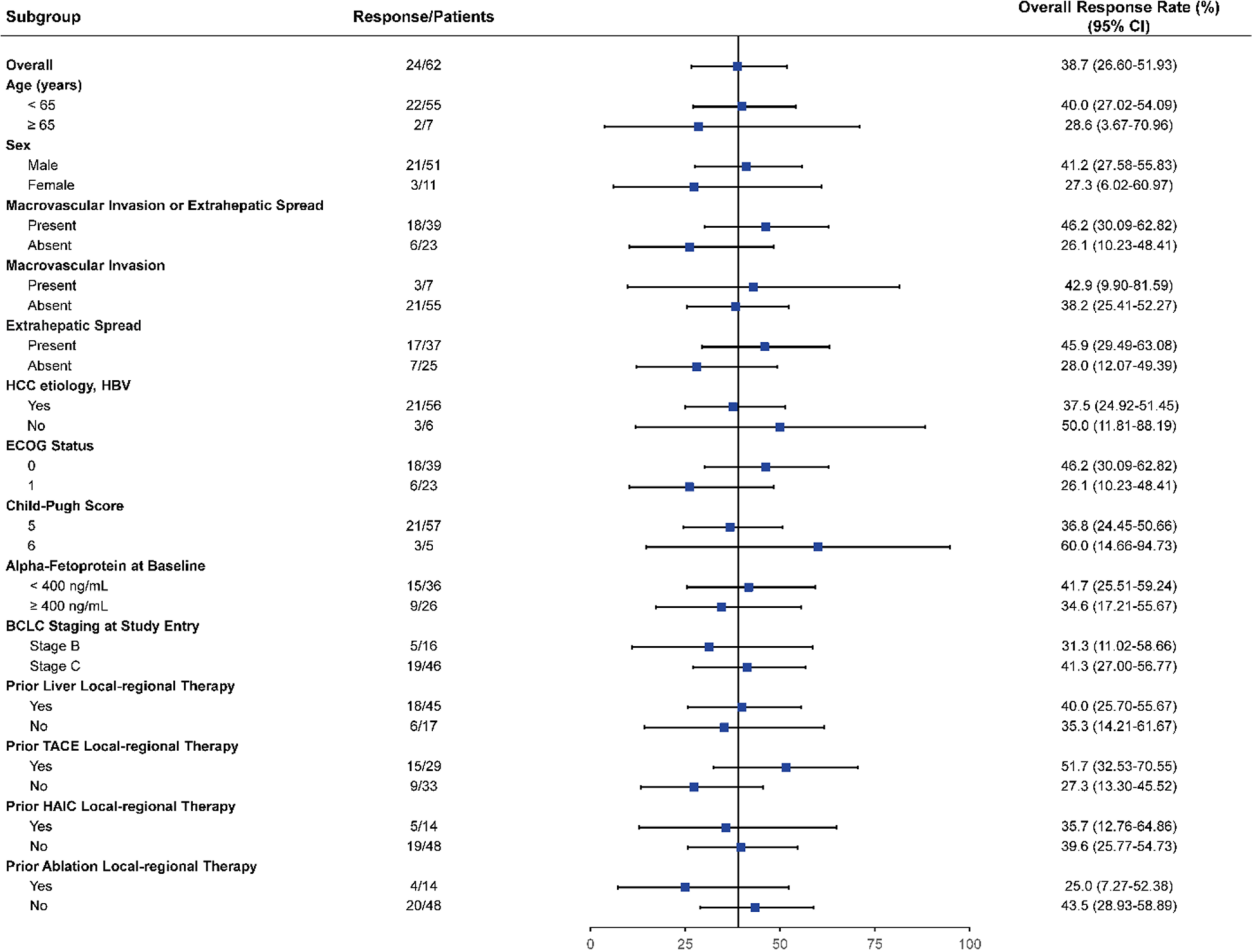
Subgroup analysis of overall response rate per RECIST v1.1 by IRC review (*N* = 62). IRC = Independent Review Committee, RECIST = Response Evaluation Criteria in Solid Tumors, HBV = hepatitis B virus, ECOG PS = Eastern Cooperative Oncology Group Performance Score, BCLC = Barcelona Clinic Liver Cancer, TACE = transarterial chemoembolization, HAIC = hepatic arterial infusion chemotherapy, CI = confidence interval. Macrovascular Invasion or Extrahepatic Spread included 2 macrovascular invasion only patients, 32 extrahepatic spread only patients and 5 patients had both macrovascular invasion and extrahepatic spread

Of the total 62 patients in the EAS, confirmed ORR by IRC assessment for the primary endpoint was 38.7% (95% CI, 26.6–51.9) with 24 responders per RECIST v1.1. Confirmed ORR by IRC assessment was 46.8% (95% CI, 34.0–59.9) per mRECIST, and 38.7% (95% CI, 26.6–51.9) per iRECIST. The confirmed ORR assessed by investigators per RECIST v1.1, mRECIST and iRECIST were 41.9% (95% CI, 29.5–55.2), 46.8% (95% CI, 34.0–59.9), and 43.5% (95% CI, 31.0–56.7), respectively (Table 2). Reductions in tumor size of target lesions per RECIST v1.1 were reported in 72.6% (*n* = 45) of patients by IRC and 80.6% (*n* = 50) by investigator assessment (Fig. 1). The subgroup analysis of IRC assessed ORRs per RECIST v1.1 showed that the study treatment performed equally in patients with different prognostic features (Fig. 2).

**Table 2:** Tumor response by IRC and investigator review per RECIST v1.1, mRECIST and iRECIST (EAS, *N* = 62)

	**IRC review**			**Investigator review**		
	**RECIST v1.1**	**mRECIST**	**iRECIST**	**RECIST v1.1**	**mRECIST**	**iRECIST**
Confirmed objective response, n (%) [95% CI^a^]	24 (38.7) [26.6, 51.9]	29 (46.8) [34.0, 59.9]	24 (38.7) [26.6, 51.9]	26 (41.9) [29.5, 55.2]	29 (46.8) [34.0, 59.9]	27 (43.5) [31.0, 56.7]
BOR/iBOR, n (%)						
CR/iCR	0 (0.0)	0 (0.0)	0 (0.0)	1 (1.6)	1 (1.6)	1 (1.6)
PR/iPR	24 (38.7)	29 (46.8)	24 (38.7)	25 (40.3)	28 (45.2)	26 (41.9)
SD/iSD	32 (51.6)	27 (43.5)	32 (51.6)	27 (43.5)	24 (38.7)	28 (45.2)
PD	5 (8.1)	5 (8.1)	n/a	8 (12.9)	8 (12.9)	n/a
iUPD	n/a	n/a	2 (3.2)	n/a	n/a	2 (3.2)
iCPD	n/a	n/a	3 (4.8)	n/a	n/a	4 (6.5)
Not assessable^b^	1 (1.6)	1 (1.6)	1 (1.6)	1 (1.6)	1 (1.6)	1 (1.6)
DCR, n (%) [95% CI^a^]	56 (90.3) [80.1, 96.4]	56 (90.3) [80.1, 96.4]	56 (90.3) [80.1, 96.4]	53 (85.5) [74.2, 93.1]	53 (85.5) [74.2, 93.1]	55 (88.7) [78.1, 95.3]

^a^95% CI was estimated using the Clopper-Pearson method. ^b^ One patient received 1 dose of tislelizumab and 8 days lenvatinib and died with confirmed clinical disease progression before the first radiological assessment. *IRC* Independent Review Committee, *RECIST* Response Evaluation Criteria in Solid Tumors, *mRECIST* Modified RECIST, *BOR* Best overall response, *CR* Complete response, *PR* Partial response, *PD* Progressive disease, *SD* Stable disease, *iRECIST* *Immune RECIST* “i” indicates immune responses assessed using iRECIST, iBOR = BOR, iCR = CR iPR = PR iSD = SD, *iUPD* Unconfirmed disease progression, *iCPD* Confirmed disease progression, *DCR* Disease control rate, *EAS* Efficacy evaluable analysis set, *CI* Confidence interval, *n/a* Not applicable, *NE* Not evaluable

Median DoR per RECIST v1.1 was not reached by either IRC or investigator review, with the 6-month event-free rate of 88.9% (95% CI, 62.4–97.1) and 72.3% (95% CI, 50.4–85.7), respectively. Twelve-month event-free rate per RECIST v1.1 was not reached either by IRC or investigator review. DCR per RECIST v1.1 was 90.3% (95% CI, 80.1–96.4) by IRC and 85.5% (95% CI, 74.2–93.1) by investigator review (Table 2).

**Fig. 3:**
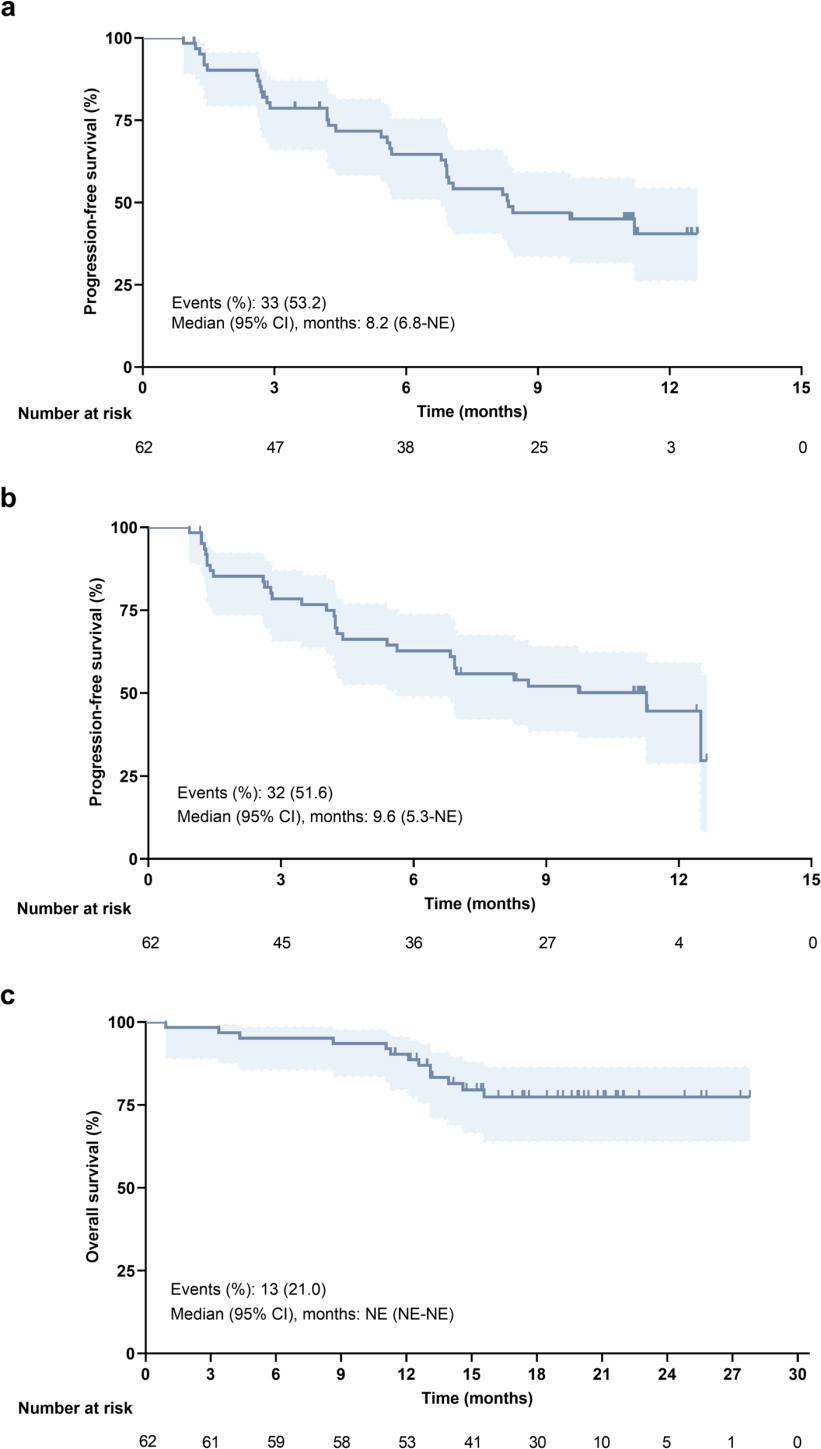
Kaplan–Meier plots for PFS per RECIST v1.1 and OS (*N* = 62). **a** PFS by IRC review; **b** PFS by investigator review; **c** OS. PFS = progression-free survival, OS = overall survival, IRC = independent review committee, CI = confidence interval, NE = not estimable

At the data cutoff, median PFS per RECIST v1.1 was 8.2 months (95% CI, 6.8–not estimable [NE]) by IRC and 9.6 months (95% CI, 5.3–NE) by investigator review. PFS rates at 12 months were 40.5% (95% CI, 26.2–54.2) and 43.7% (95% CI, 28.3–58.1), respectively (Fig. 3a-b). Median OS was not reached (Fig. 3c). The 6-month and 12-month OS rates were 95.2% (95% CI, 85.7–98.4) and 88.6% (95% CI, 77.7–94.4), respectively. PFS per mRECIST and iRECIST assessed by IRC or investigator review is shown in Additional file 1: Fig. S3.

### Safety and tolerability

All the 64 patients experienced at least one treatment-emergent adverse events (TEAEs), with 22 (34.4%) experiencing TEAEs of grade ≥ 3 (Additional file 3: Table S2). And 95.3% of patients reported ≥ 1 treatment-related adverse events (TRAEs); TRAEs reported in ≥ 20% of patients included proteinuria (46.9%), hypertension (35.9%), hypothyroidism (31.3%), aspartate aminotransferase increased (26.6%), platelet count decreased (23.4%), weight decreased (23.4%), and palmar-plantar erythrodysesthesia syndrome (20.3%) (Table 3). Grade ≥ 3 TRAEs occurred in 18 (28.1%) patients.

**Table 3:** Frequency of AEs (*N* = 64)

	**Any grade**	**Grade 3**	**Grade 4**
**TRAEs**			
Any TRAEs	61 (95.3)	14 (21.9)	3 (4.7)
Proteinuria	30 (46.9)	0 (0.0)	0 (0.0)
Hypertension	23 (35.9)	2 (3.1)	0 (0.0)
Hypothyroidism	20 (31.3)	0 (0.0)	0 (0.0)
Aspartate aminotransferase increased	17 (26.6)	0 (0.0)	0 (0.0)
Platelet count decreased	15 (23.4)	4 (6.3)	0 (0.0)
Weight decreased	15 (23.4)	0 (0.0)	0 (0.0)
Palmar-plantar erythrodysaesthesia syndrome	13 (20.3)	4 (6.3)	0 (0.0)
Lipase increased	12 (18.8)	2 (3.1)	1 (1.6)
Amylase increased	10 (15.6)	1 (1.6)	0 (0.0)
Blood creatine phosphokinase MB increased	10 (15.6)	0 (0.0)	0 (0.0)
Blood lactate dehydrogenase increased	10 (15.6)	0 (0.0)	0 (0.0)
Blood bilirubin increased	9 (14.1)	0 (0.0)	0 (0.0)
Rash	8 (12.5)	0 (0.0)	0 (0.0)
White blood cell count decreased	7 (10.9)	1 (1.6)	0 (0.0)
Haematuria	7 (10.9)	0 (0.0)	0 (0.0)
Diarrhoea	7 (10.9)	0 (0.0)	0 (0.0)
Dysphonia	7 (10.9)	0 (0.0)	0 (0.0)
**imAEs**			
Any imAEs	30 (46.9)	3 (4.7)	0 (0.0)
Hypothyroidism	20 (31.3)	0 (0.0)	0 (0.0)
Rash	8 (12.5)	0 (0.0)	0 (0.0)
Hyperthyroidism	3 (4.7)	0 (0.0)	0 (0.0)
Myositis	2 (3.1)	0 (0.0)	0 (0.0)
Pancreatitis	2 (3.1)	2 (3.1)	0 (0.0)
Thyroiditis	2 (3.1)	0 (0.0)	0 (0.0)
Adrenal insufficiency	1 (1.6)	0 (0.0)	0 (0.0)
Enterocolitis	1 (1.6)	1 (1.6)	0 (0.0)

Data are presented as n (%). TRAEs at any grade occurring in ≥ 10% of patients are listed. No grade 5 adverse events occurred among TRAEs reported at an overall frequency of ≥ 10%. imAEs occurring in ≥ 1 patient. Data are listed in order of decreased frequency of any grade TRAE. *AEs* Adverse events, *TRAEs* Treatment-related adverse events, *imAEs* Immune-mediated adverse events

Serious adverse events (SAEs) were reported in 11 (17.2%) patients; 7 (10.9%) experienced treatment-related SAEs. During the study, 3 (4.7%) deaths occurred. Two deaths were reported as unrelated to study treatments. One death was reported as treatment related. This patient received one dose of tislelizumab and 8 days of lenvatinib and died due to respiratory failure. Death was attributed to disease progression and study treatment. TRAEs leading to treatment discontinuation occurred in 3 (4.7%) patients. TRAEs led to treatment modification in 35 (54.7%) patients.

Immune-mediated adverse events (imAEs) occurred in 30 (46.9%) patients, and most were grade 1 or 2 in severity. Three (4.7%) patients with at least one imAEs received systemic corticosteroids. Grade 3 imAEs occurred in 3 (4.7%) patients (pancreatitis [*n* = 2, 3.1%]; enterocolitis [*n* = 1, 1.6%]) (Table 3). imAEs at grade 4 or higher were not observed. imAEs led to tislelizumab interruption in 7 (10.9%) patients and tislelizumab discontinuation in 1 (1.6%) patient.

## Discussion

In this open-label, multi-center, phase 2 trial, the combination of tislelizumab and lenvatinib demonstrated promising clinical efficacy in patients with uHCC who had received no prior systemic therapy. The study showed statistical superiority of tislelizumab plus lenvatinib compared with historical data of the lenvatinib arm from the phase 3 REFLECT study [5] in the first-line setting in uHCC patients, with a confirmed ORR of 38.7% per RECIST v1.1 by IRC review. Objective response was observed across subgroups. After a median follow-up duration of 15.7 months, tislelizumab plus lenvatinib yielded a promising median PFS (8.2 months; 95% CI, 6.8–NE) per RECIST v1.1 by IRC assessment. The median OS was not reached, and 1-year OS rate was 88.6% (95% CI, 77.7–94.4). The combination was generally well tolerated, and the safety profile was consistent with tislelizumab or lenvatinib administered alone.

The IMbrave 150 study demonstrated atezolizumab combined with bevacizumab resulted in superior OS and PFS outcomes compared with sorafenib, supported by an improved ORR of 30% and a DCR of 74% by RECIST 1.1 with a median DoR of 18.1 months [15]. Current study revealed a confirmed ORR of 38.7% for tislelizumab and lenvatinib combination therapy, with a DCR of 90.3% and a durable DoR (not reached). These results were similar to the results observed in studies of other combinations of ICIs with TKIs in the first-line setting (Additional file 3: Table S3) [9, 14–16], such as CARES-310 (camrelizumab plus rivoceranib: ORR 25%, DCR 78%) [16] and LEAP-002 (pembrolizumab plus lenvatinib: ORR 26.1%, DCR 81.3%) [9]. Median PFS was 8.2 months per RECIST v1.1 by IRC and 9.6 months by investigator review in current study, which was similar with that reported in large phase 3 trials, with PFS ranging from 5.6 to 8.2 months [9, 16, 17]. The efficacy of tislelizumab plus lenvatinib was close to previous phase 3 trials of other combinations as first-line therapy for uHCC.

In this study, the combination therapy of tislelizumab plus lenvatinib was generally well tolerated with no new or unexpected toxicities. The combination showed safety profile consistent with profiles of each individual agent as reported in previous studies [5, 13, 18], and with other combinations with ICIs and anti-VEGFR antibodies or targeted therapies (Additional file 3: Table S4) [9, 14–16]. Notably, grade ≥ 3 TRAEs occurred in only 28.1% of patients with study treatment, which was numerically lower than the rates observed with other combinations (atezolizumab plus bevacizumab in IMbrave 150, 43% [15]; pembrolizumab plus lenvatinib in LEAP-002, 63% [9]; camrelizumab plus rivoceranib in CARES-310, 81% [16]), indicating that tislelizumab plus lenvatinib may present a favourable safety profile. However, it is important to interpret these results with caution considering the different duration of treatment, the limited sample size of the current study and indirect comparison.

While LEAP-002 did not demonstrate statistical superiority of pembrolizumab with lenvatinib over lenvatinib alone, combination treatment led to numerically better results in key endpoints, especially in the subgroup of patients with HBV etiology, which supports clinical value of ICI plus TKI combination in treatment of uHCC. Designing the current study, it was assumed that tislelizumab plus lenvatinib combination might provide larger OS benefit than tislelizumab monotherapy. In this study, the 12-month OS rate was 88.6% (median OS, not reached), which was numerically higher than that from RATIONALE 301 Chinese population (56.6%) [19], indicated that there might be a trend towards improved OS rate with this combination. Taken together, these results indicated that tislelizumab plus lenvatinib could be a promising therapeutic option in the first-line treatment of uHCC with encouraging efficacy and tolerability.

In addition to RECIST v1.1 and mRECIST, we also utilized iRECIST as a method to assess tumor response in this study. This approach allows for the identification of atypical responses, such as delayed responses that may occur after pseudoprogression, thereby potentially preventing early treatment discontinuation due to pseudoprogression [20]. In this study, 6 patients who remained on study treatment after iUPD had target lesions shrinkage compared with baseline and had no new lesions, of which 1 received curative surgery subsequently, suggesting that the utilization of iRECIST may benefit some patients by preventing early withdrawal from a potentially effective treatment.

The study has several limitations. Firstly, although the study was designed based on predefined statistical assumptions and sample size calculation, the conclusion on the superiority of the combination than lenvatinib monotherapy from REFLECT study needs cautions based on its single-arm design. Though the latest data of lenvatinib arm from LEAP-002 study demonstrated longer OS and PFS than REFLECT, the ORR was similar, and REFLECT study was the only phase 3 study could be referred as a historical control when the present study was designed. Secondly, the design of maximum treatment period of 12 months might be insufficient for patients who still presenting controlled disease. However, in this study, the continued use of tislelizumab was allowed for patients who completed 12 months of treatment without iCPD and were deemed to still benefit from the study treatment based on investigator assessment. Thirdly, the study only included patients from China, with a quite large proportion (90.6%) of HBV- related HCC. Further verification of the benefits of the combination of tislelizumab plus lenvatinib for HCC patients is warranted in large-scale controlled studies including patients of other etiology.

## Conclusions

This phase 2 trial demonstrated that the combination of tislelizumab and lenvatinib was statistically superior compared with historical ORR data of lenvatinib in the first-line treatment of uHCC patients. This combination led to promising PFS and OS rates and was generally well tolerated.

### Supplementary Information

**Additional file 1:** **Fig. S1.** Study design. **Fig. S2.** Patient flow diagram. **Fig. S3.** Kaplan-Meier plots for PFS per mRECIST and iRECIST by IRC and investigator review.

**Additional file 2.** Design of safety run-in. Assessment of Dose-Limiting Toxicity. Definition of Dose-Limiting Toxicity. R code (R 4.1.2) to calculate the sample size based on Simon’s 2 design.

**Additional file 3:** **Table S1.** Subsequent anticancer medications or cancer-related procedures/surgery during survival follow-up (*N*=64). **Table S2.** Summary of adverse events (*N*=64). **Table S3.** Key efficacy data of trials for the first-line treatment of HCC. **Table S4.** Summary of TEAEs and imAEs data of trials for the first-line treatment of HCC.

## Data Availability

All data relevant to the study are included in this article and its additional files. Further inquiries can be directed to the corresponding author.

## Abbreviations

AEs: Adverse events
AFP: Alpha-fetoprotein
BCLC: Barcelona Clinic Liver Cancer
BOR: Best overall response
CR: Complete response
CI: Confidence interval
DCR: Disease control rate
DLTs: Dose-limiting toxicities
DoR: Duration of response
EAS: Efficacy evaluable analysis set
ECOG: Eastern Cooperative Oncology Group
ECOG PS: Eastern Cooperative Oncology Group Performance Score
HAIC: Hepatic arterial infusion chemotherapy
HBV: Hepatitis B virus
HCC: Hepatocellular carcinoma
HR: Hazard ratio
ICIs: Immune-checkpoint inhibitors
iCPD: Immune confirmed disease progression
imAEs: Immune-mediated adverse events
IRC: Independent review committee
iRECIST: Immune Response Evaluation Criteria in Solid Tumors
iUPD: Immune unconfirmed disease progression
MedDRA: Medical Dictionary for Regulatory Activities
n/a: Not applicable
NCI-CTCAE: National Cancer Institute Common Terminology Criteria for Adverse Events
NE: Not estimable
ORRs: Objective response rates
OS: Overall survival
PD: Disease progression
PFS: Progression-free survival
PR: Partial response
RECIST: Response Evaluation Criteria in Solid Tumors
SAEs: Serious adverse events
SAS: Safety analysis set
SD: Stable disease
TACE: Transarterial chemoembolization
TE: Transarterial embolism
TEAEs: Treatment-emergent adverse events
TKIs: Tyrosine kinase inhibitors
TRAEs: Treatment-related adverse events
uHCC: Unresectable hepatocellular carcinoma

## References

[1] Global Cancer Observatory. Cancer Today. All cancers fact sheet. https://gco.iarc.fr/today/data/factsheets/cancers/39-All-cancers-fact-sheet.pdf. Accessed 21 Feb 2023.

[2] Singal AG, Lampertico P, Nahon P (2020). Epidemiology and surveillance for hepatocellular carcinoma: New trends. J Hepatol.

[3] Llovet JM, Kelley RK, Villanueva A, Singal AG, Pikarsky E, Roayaie S (2021). Hepatocellular carcinoma Nat Rev Dis Primers.

[4] Llovet JM, Ricci S, Mazzaferro V, Hilgard P, Gane E, Blanc JF (2008). Sorafenib in advanced hepatocellular carcinoma. N Engl J Med.

[5] Kudo M, Finn RS, Qin S, Han KH, Ikeda K, Piscaglia F (2018). Lenvatinib versus sorafenib in first-line treatment of patients with unresectable hepatocellular carcinoma: a randomised phase 3 non-inferiority trial. Lancet.

[6] Fukumura D, Kloepper J, Amoozgar Z, Duda DG, Jain RK (2018). Enhancing cancer immunotherapy using antiangiogenics: opportunities and challenges. Nat Rev Clin Oncol.

[7] Kato Y, Tabata K, Kimura T, Yachie-Kinoshita A, Ozawa Y, Yamada K (2019). Lenvatinib plus anti-PD-1 antibody combination treatment activates CD8+ T cells through reduction of tumor-associated macrophage and activation of the interferon pathway. PLoS ONE.

[8] Kimura T, Kato Y, Ozawa Y, Kodama K, Ito J, Ichikawa K (2018). Immunomodulatory activity of lenvatinib contributes to antitumor activity in the Hepa1-6 hepatocellular carcinoma model. Cancer Sci.

[9] Llovet JM, Kudo M, Merle P, Meyer T, Qin S, Ikeda M (2023). Lenvatinib plus pembrolizumab versus lenvatinib plus placebo for advanced hepatocellular carcinoma (LEAP-002): a randomised, double-blind, phase 3 trial. Lancet Oncol.

[10] Qin SK et al. First-line lenvatinib plus pembrolizumab for advanced heptocellular carcinoma: LEAP-002 asian subgroup analysis. JSMO, 2023.

[11] Yang X, Chen B, Wang Y, Wang Y, Long J, Zhang N, et al. Real-world efficacy and prognostic factors of lenvatinib plus PD-1 inhibitors in 378 unresectable hepatocellular carcinoma patients. Hepatol Int. 2023:1–11. 10.1007/s12072-022-10480-y PMC9907200 36753026

[12] Zhang T, Song X, Xu L, Ma J, Zhang Y, Gong W (2018). The binding of an anti-PD-1 antibody to FcγRΙ has a profound impact on its biological functions. Cancer Immunol Immunother.

[13] Qin S, Kudo M, Meyer T, Bai Y, Guo Y, Meng Z (2023). Tislelizumab vs sorafenib as first-line treatment for unresectable hepatocellular carcinoma: a phase 3 randomized clinical trial. JAMA Oncol.

[14] Finn RS, Ikeda M, Zhu AX, Sung MW, Baron AD, Kudo M (2020). Phase Ib Study of Lenvatinib Plus Pembrolizumab in Patients With Unresectable Hepatocellular Carcinoma. J Clin Oncol.

[15] Cheng A-L, Qin S, Ikeda M, Galle PR, Ducreux M, Kim T-Y, et al. Updated efficacy and safety data from IMbrave150: Atezolizumab plus bevacizumab vs. sorafenib for unresectable hepatocellular carcinoma. J Hepatol. 2022;76:862–73. 10.1016/j.jhep.2021.11.030 34902530

[16] Qin S, Chan SL, Gu S, Bai Y, Ren Z, Lin X (2023). Camrelizumab plus rivoceranib versus sorafenib as first-line therapy for unresectable hepatocellular carcinoma (CARES-310): a randomised, open-label, international phase 3 study. Lancet.

[17] Ren Z, Xu J, Bai Y, Xu A, Cang S, Du C (2021). Sintilimab plus a bevacizumab biosimilar (IBI305) versus sorafenib in unresectable hepatocellular carcinoma (ORIENT-32): a randomised, open-label, phase 2–3 study. Lancet Oncol.

[18] Ren Z, Ducreux M, Abou-Alfa GK, Merle P, Fang W, Edeline J (2023). Tislelizumab in patients with previously treated advanced hepatocellular carcinoma (RATIONALE-208): a multicenter, non-randomized, open-label, phase 2 trial. Liver Cancer.

[19] Qin S, Guo Y, Meng Z, Wu J, Gu K, Zhang T (2022). LBA2 Tislelizumab (TIS) versus sorafenib (SOR) in first-line (1L) treatment of unresectable hepatocellular carcinoma (HCC): The RATIONALE-301 Chinese subpopulation analysis. Ann Oncol.

[20] Seymour L, Bogaerts J, Perrone A, Ford R, Schwartz LH, Mandrekar S (2017). iRECIST: guidelines for response criteria for use in trials testing immunotherapeutics. Lancet Oncol.

