# Supplementary material for: Efficacy and safety of tislelizumab plus lenvatinib as first-line treatment in patients with unresectable hepatocellular carcinoma: a multicenter, single-arm, phase 2 trial
Source: BMC Med. 2024 Apr 23;22:172. doi: 10.1186/s12916-024-03356-5 (PMC11036623; doi:10.1186/s12916-024-03356-5)
Supplement: Supplementary file 1 — Additional file 1: Fig. S1. Study design. Fig. S2. Patient flow diagram. Fig. S3. Kaplan-Meier plots for PFS per mRECIST and iRECIST by IRC and investigator review. [file 12916_2024_3356_MOESM1_ESM.docx]

**Additional File 1**

**Contents:**

[**Supplemental figures** 2](#_Toc160801958)

[**Fig. S1. Study design** 2](#_Toc160801959)

[**Fig. S2. Patient flow diagram** 3](#_Toc160801960)

[**Fig. S3. Kaplan-Meier plots for PFS per mRECIST and iRECIST by IRC and investigator review.** 4](#_Toc160801961)

# **Supplemental figures**

## **Fig. S1. Study design**


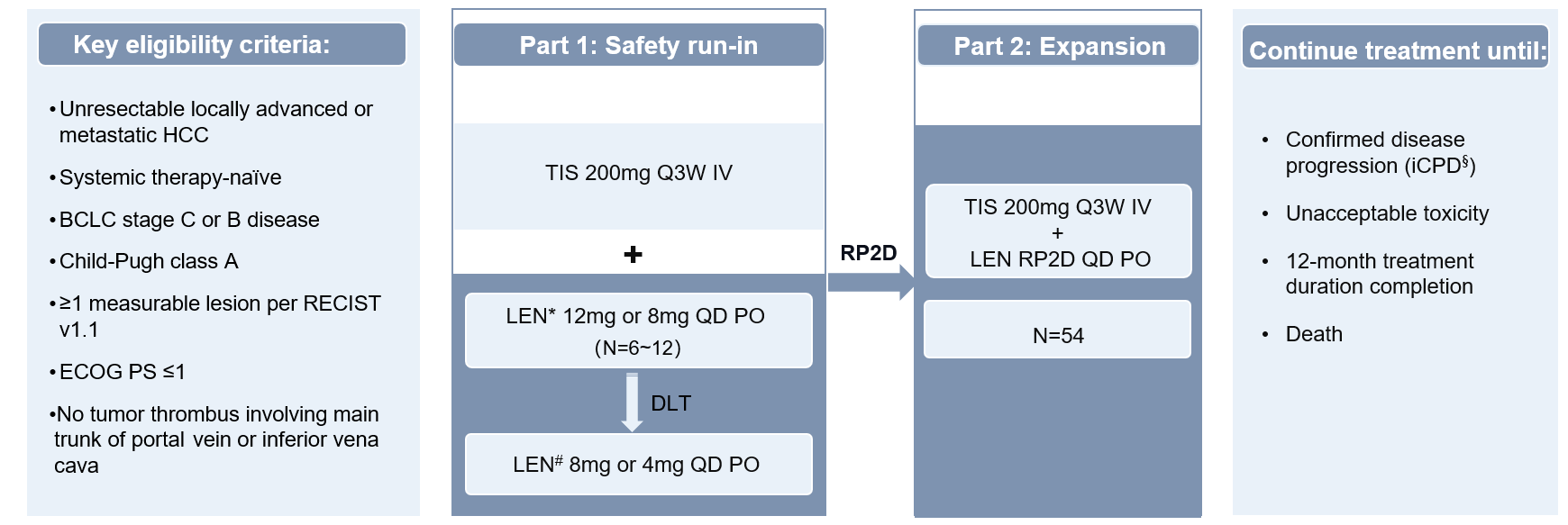


^*^Starting dose: 12 mg (body weight ≥60 kg) or 8mg (body weight <60 kg). ^#^Reduced dose: 8mg (body weight ≥60 kg) or 4mg (body weight <60 kg). ^§^ assessed by iRECIST. BCLC=Barcelona Clinic Liver Cancer, DLT=dose limiting toxicity, ECOG PS=Eastern Cooperative Oncology Group performance score, HCC=hepatocellular carcinoma, iRECIST=immune Response Evaluation Criteria in Solid Tumors, TIS=tislelizumab, LEN=lenvatinib, PO=orally, Q3W=every three weeks, QD=once a day, RP2D=recommended phase 2 dose


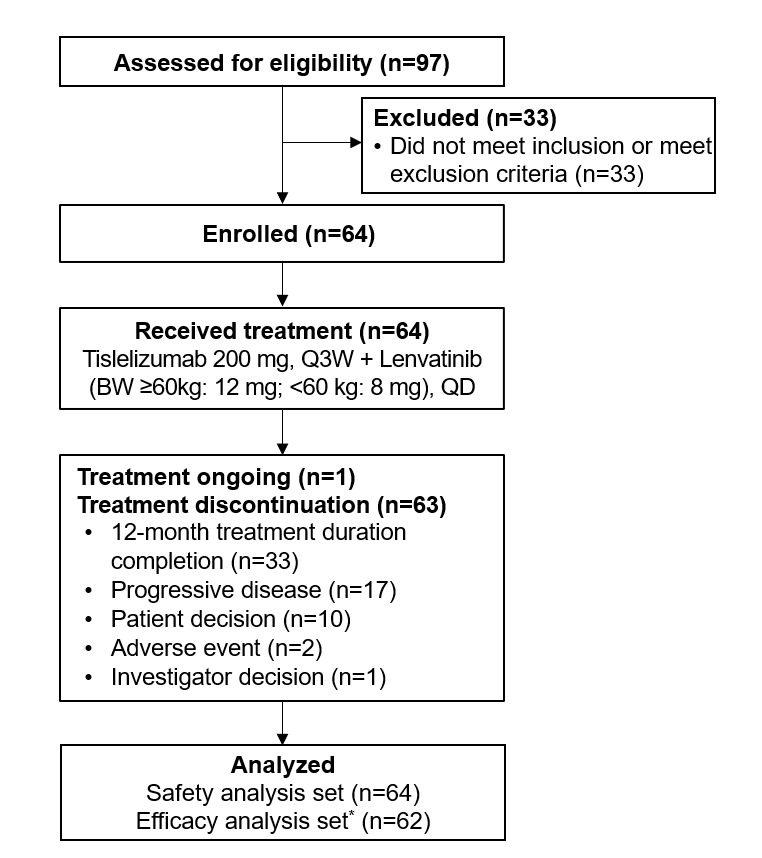


## **Fig. S2. Patient flow diagram**

^*^The first 60 patients' data from efficacy analysis set were used for statistical superiority test. BW=body weight, Q3W=every three weeks, QD=once a day.


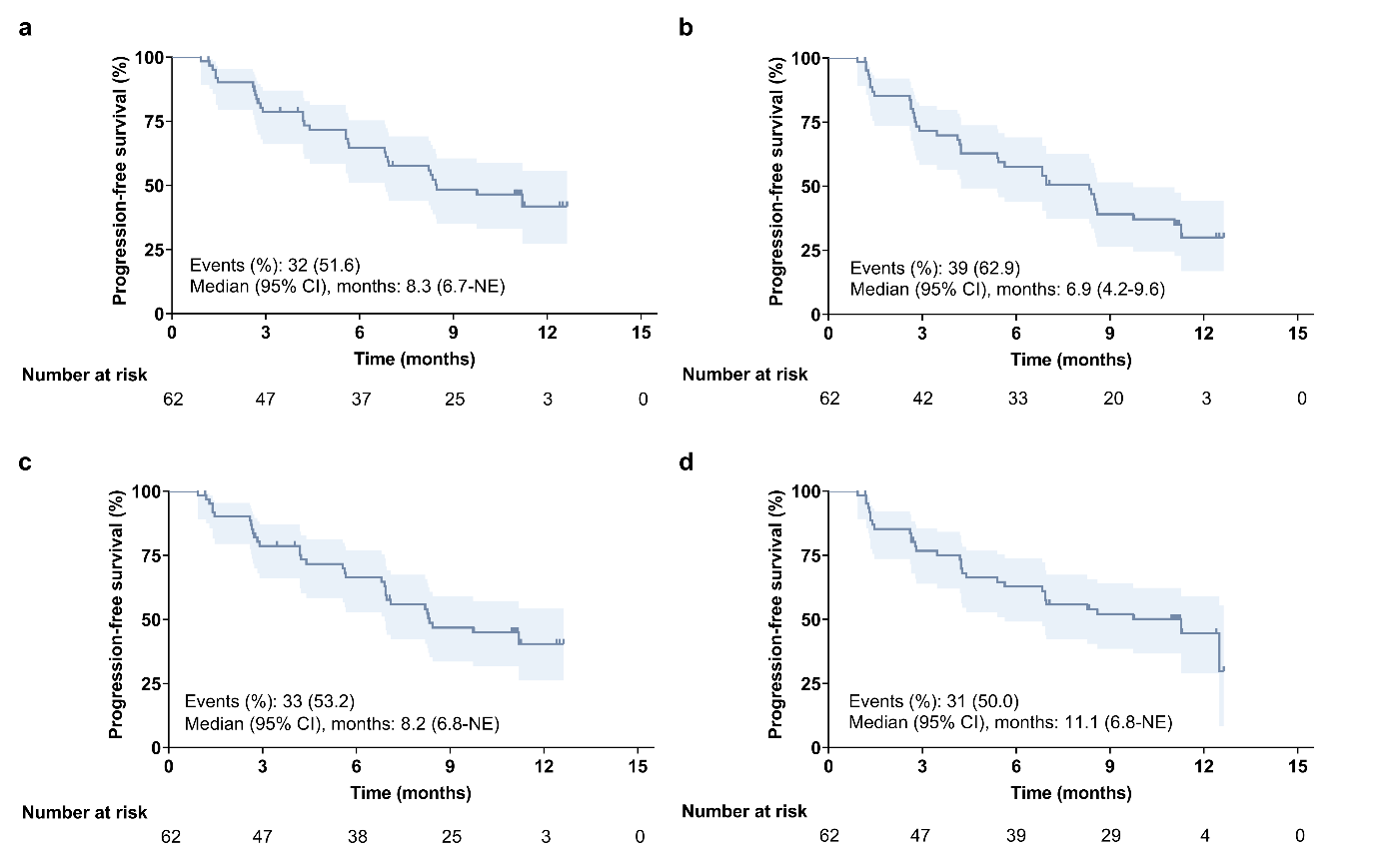


## **Fig. S3. Kaplan-Meier plots for PFS per mRECIST and iRECIST by IRC and investigator review.**

PFS per mRECIST assessed by (a) IRC and (b) investigator; PFS per iRECIST assessed by (c) IRC and (d) investigator.PFS=progression-free survival; IRC=independent review committee; RESCIST=Response Evaluation Criteria in Solid Tumors, mRECIST=modified RECIST; iRECIST=immune RECIST; CI=confidence interval, NE=not estimable.
