# Supplementary material for: Efficacy and safety of tislelizumab plus lenvatinib as first-line treatment in patients with unresectable hepatocellular carcinoma: a multicenter, single-arm, phase 2 trial
Source: BMC Med. 2024 Apr 23;22:172. doi: 10.1186/s12916-024-03356-5 (PMC11036623; doi:10.1186/s12916-024-03356-5)

**Additional File 2**

**Contents:**

[**Supplemental methods** 2](#_Toc160801071)

[**Design of safety run-in** 2](#_Toc160801072)

[**Assessment of Dose-Limiting Toxicity** 2](#_Toc160801073)

[**Definition of Dose-Limiting Toxicity** 2](#_Toc160801074)

[**R code (R 4.1.2) to calculate the sample size based on Simon’s 2 design** 4](#_Toc160801075)

# **Supplemental methods**

## **Design of safety run-in**

During safety run-in phase, 6-12 patients with unresectable locally advanced or metastatic HCC would be enrolled. Three patients with baseline body weight ≥60 kg and 3 patients with baseline body weight <60 kg would be enrolled. If ≤1 of 6 patients experienced a DLT, this dose level would be used in the expansion phase. If >1 of 6 patients experienced a DLT, the principal investigator would decide to terminate the study or to start another 6 cases to investigate lenvatinib at reduced dose [8 mg (body weight ≥60 kg) or 4 mg (body weight <60 kg)]. If ≤1 of 6 patients experienced a DLT in lenvatinib dose reduced cohort, this reduced dose level would be used in the expansion phase. If＞1 of 6 patients experienced a DLT in lenvatinib dose reduced cohort, the safety run-in phase would be stopped, and the expansion phase wouldn’t be proceeded. The safety monitoring committee (SMC) would determine whether to continue the study based on all available safety data.

## **Assessment of Dose-Limiting Toxicity**

Dose-limiting toxicity (DLT) was assessed among evaluable patients within 21 days after the first dose of study drugs. For dose reduction decision, only DLTs occurring within 21 days would be evaluated. The following patients would not be considered evaluable for DLT:

- Patients who withdrew or were withdrawn from the study before completing the DLT assessment window for reasons other than a DLT.
- Patients receiving combination therapy who did not receive ≥75% of scheduled lenvatinib and ≥67% (approximately two-thirds) of scheduled tislelizumab during the DLT assessment window unless they experienced a DLT.

## **Definition of Dose-Limiting Toxicity**

A DLT was defined as any of the following toxicities occurring during the DLT assessment window and considered by the investigator to be related to lenvatinib and/or tislelizumab.

Hematologic

- Grade 4 neutropenia lasting >7 days
- ≥Grade 3 febrile neutropenia
- Grade 3 thrombocytopenia with clinically significant bleeding
- Grade 4 thrombocytopenia lasting >3 days and requiring transfusion, or any decreased platelet count <15,000/mm^3^ or <15.0 x 10^9^/L
- ≥Grade 4 anaemia

Non-Hematologic

- ≥Grade 4 toxicity
- Grade 3 toxicity that was clinically significant and did not resolve to baseline or ≤Grade 1 within 7 days of initiating optimal supportive care

Note: The following AEs would not be considered DLTs:

- Grade 3 endocrinopathy that was adequately controlled by hormonal replacement;
- Grade 3 of tumor flare (defined as local pain, irritation, or rash localized at sites of known or suspected tumors);
- Grade 3 rash;
- Grade 3 infusion-related AE that was transient (resolving within 6 hours of onset);
- Grade 3 hypertension that was resolving within 7 days of optimal supportive care;

Note: Hypertension would be graded using NCI-CTCAE v 5.0, based on blood pressure measurements only (and not on the number of antihypertensive medications).

- Clinically insignificant or transient abnormal laboratory findings, including but not limited to following:
  - Grade 3-4 alanine aminotransferase elevation, aspartate aminotransferase elevation or hyperbilirubinemia without significant related clinical symptoms and judged by investigators and medical monitors as non-fatal risk;
  - Grade 3-4 hyperamylasaemia or hyperlipaemia that was not associated with symptoms or clinical manifestations of pancreatitis and judged by investigators and medical monitors as non-fatal risk;
  - Grade 3 proteinuria that was resolving within 7 days of optimal supportive care.

All available safety data, including AEs, and laboratory assessment would be reviewed with input from other functional representatives as appropriate.

A safety monitoring committee (SMC) would be established consisting of the sponsor’s clinical, safety, and medical team representatives (eg, medical monitor, clinical pharmacology, statistician, and drug safety) and investigators. A planned formal SMC review of safety data would be performed after at least 6 evaluable patients had completed 21-day DLT assessment in each lenvatinib dose level. The SMC would monitor the preliminary safety of lenvatinib in combination with tislelizumab and determined whether to continue the study based on the available data (eg, safety data) in this study.

## **R code (R 4.1.2) to calculate the sample size based on Simon’s 2 design**


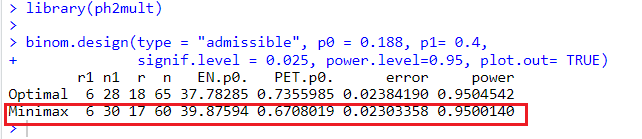

Supplement: Supplementary file 2 — Additional file 2. Design of safety run-in. Assessment of Dose-Limiting Toxicity. Definition of Dose-Limiting Toxicity. R code (R 4.1.2) to calculate the sample size based on Simon’s 2 design. [file 12916_2024_3356_MOESM2_ESM.docx]
