# Supplementary material for: Efficacy and safety of tislelizumab plus lenvatinib as first-line treatment in patients with unresectable hepatocellular carcinoma: a multicenter, single-arm, phase 2 trial
Source: BMC Med. 2024 Apr 23;22:172. doi: 10.1186/s12916-024-03356-5 (PMC11036623; doi:10.1186/s12916-024-03356-5)
Supplement: Supplementary file 3 — Additional file 3: Table S1. Subsequent anticancer medications or cancer-related procedures/surgery during survival follow-up (N=64). Table S2. Summary of adverse events (N=64). Table S3. Key efficacy data of trials for the first-line treatment of HCC. Table S4. Summary of TEAEs and imAEs data of trials for the first-line treatment of HCC. [file 12916_2024_3356_MOESM3_ESM.docx]

**Additional File 3**

**Contents:**

[**Supplemental tables** 2](#_Toc160801271)

[**Table S1. Subsequent anticancer medications or cancer-related procedures/surgery during survival follow-up (N=64)** 2](#_Toc160801273)

[**Table S2. Summary of adverse events (N=64)** 3](#_Toc160801274)

[**Table S3. Key efficacy data of trials for the first-line treatment of HCC** 5](#_Toc160801275)

[**Table S4. Summary of TEAEs and imAEs data of trials for the first-line treatment of HCC** 6](#_Toc160801276)

# **Supplemental** **tables**

## **Table S1. Subsequent anticancer medications or cancer-related procedures/surgery during survival follow-up (N=64)**

|  | **Total (N=64)**  **n (%)** |
| --- | --- |
| **Patients with any subsequent systemic anticancer therapy** | 23 (35.9) |
| Chemotherapy | 2 (3.1) |
| Any immunotherapy | 6 (9.4) |
| Targeted therapy | 15 (23.4) |
| Other therapies | 1 (1.6) |
| **Patients with any subsequent cancer-related procedure/surgery** | 17 (26.6) |
| Ablation | 5 (7.8) |
| HAIC | 4 (6.3) |
| Liver Transplantation | 1 (1.6) |
| Radio Therapy | 3 (4.7) |
| Surgery | 5 (7.8) |
| TACE | 4 (6.3) |
| Other | 6 (9.4) |

HAIC=hepatic arterial infusion chemotherapy, TACE=transarterial chemoembolization.

## **Table S2. Summary of adverse events (N=64)**

| **AEs** | **Number of patients (%)** |
| --- | --- |
| **TEAEs** |  |
| ≥1 TEAEs | 64 (100.0) |
| Grade ≥3 | 22 (34.4) |
| Serious | 11 (17.2) |
| Led to treatment modification | 37 (57.8) |
| Tislelizumab infusion interruption | 0 |
| Tislelizumab dose delay | 19 (29.7) |
| Lenvatinib dose interruption | 28 (43.8) |
| Lenvatinib dose reduction | 23 (35.9) |
| Led to treatment discontinuation | 4 (6.3) |
| Led to death | 3 (4.7) |
| **TRAEs** |  |
| ≥1 TRAEs | 61 (95.3) |
| Grade ≥3 | 18 (28.1) |
| Serious | 7 (10.9) |
| Led to treatment modification | 35 (54.7) |
| Tislelizumab infusion interruption | 0 |
| Tislelizumab dose delay | 18 (28.1) |
| Lenvatinib dose interruption | 26 (40.6) |
| Lenvatinib dose reduction | 23 (35.9) |
| Led to treatment discontinuation | 3 (4.7) |
| Led to death | 1 (1.6) |
| **imAEs** |  |
| ≥1 imAEs | 30 (46.9) |
| Grade ≥3 | 3 (4.7) |
| Serious | 3 (4.7) |
| Led to tislelizumab modification^a^ | 7 (10.9) |
| Led to tislelizumab discontinuation | 1 (1.6) |
| Led to death | 0 |
| Treated with systemic corticosteroids | 3 (4.7) |

^a^Interrupted or delayed dose. TEAE=treatment-emergent adverse events, TRAE=treatment-related adverse events, imAE=immune-mediated adverse event

## **Table S3. Key efficacy data of trials for the first-line treatment of HCC**

| **ICIs+**  **anti-VEGFR/targeted therapy** | **Atezolizumab+**  **Bevacizumab [15]** | **Camrelizumab+Apatinib [16]** | **Pembrolizumab+Lenvatinib [9]** | **Pembrolizumab+Lenvatinib [14]** | **Tislelizumab+Lenvatinib^*^** |
| --- | --- | --- | --- | --- | --- |
| **Trial number (Phase)** | IMbrave150 (III) | CARES-310 (III) | LEAP-002 (III) | KEYNOTE-524 (Ib) | NCT 04401800 (II) |
| **Sample size (treatment arm)** | 336 | 272 | 395 | 100 | 64 |
| **Median follow-up duration, month** | 15.6 | 14.5 | 32.1 | 10.6 | 15.7 |
| **ORR, %, 95%CI** **(IRC RECIST 1.1)** | 30 (25, 35) | 25 (20, 31) | 26.1 (21.8, 30.7） | 36 (26.6, 46.2） | 38.7 (26.6, 51.9) |
| **DCR, %,** **95%CI** **(IRC RECIST 1.1)** | 74 (95%CI NA) | 78 (73, 83) | 81.3(77.1 85.0) | 88 (80.0, 93.6) | 90.3 (80.1, 96.4) |
| **Median PFS, month, 95%CI (IRC RECIST 1.1)** | 6.9 (5.7, 8.6) | 5.6 (5.5, 7.4) | 8.2 (6.3, 8.3) | 8.6 (7.1, 9.7) | 8.2 (6.8, NE) |
| **12-month OS rate, %**, **95%CI** | 67 (95%CI NA) | 76.5 (71.0, 81.1) | NA | 67.5 (56.5, 76.3) | 88.6 (77.7, 94.4) |

NA, not available. NE, not evaluable. IRC, independent review committee.

*Data from current study.

Note: For the different setting and population, these data could not be compared directly, and should be cautiously interpreted.

## **Table S4. Summary of TEAEs and imAEs data of trials for the first-line treatment of HCC**

| **Trial number** **(Phase)** | **IMbrave150**  **(III) [15]** | **CARES-310**  **(III) [16]** | | **LEAP-002**  **(III) [9]** | **KEYNOTE-524 (Ib)**  **[14]** | **NCT 04401800^*^** **(II)** |
| --- | --- | --- | --- | --- | --- | --- |
| **Treatment arm** | **Atezolizumab+Bevacizumab** | **Camrelizumab+Apatinib** | **Pembrolizumab+Lenvatinib** | | **Pembrolizumab+Lenvatinib** | **Tislelizumab+Lenvatinib** |
| **Sample size** | 329 | 272 | 395 | | 100 | 64 |
| **Any TEAEs** | 98% | 99% | NA | | 99% | 100% |
| **Any imAEs** | NA | 57% | 53% | | NA | 46.9% |

NA, not available

*Data from current study.

Note: For the different setting and population, these data could not be compared directly, and should be cautiously interpreted.
